# Assessment of a Crisis Standards of Care Scoring System for Resource Prioritization and Estimated Excess Mortality by Race, Ethnicity, and Socially Vulnerable Area During a Regional Surge in COVID-19

**DOI:** 10.1001/jamanetworkopen.2022.1744

**Published:** 2022-03-15

**Authors:** Elisabeth D. Riviello, Tenzin Dechen, Ashley L. O’Donoghue, Michael N. Cocchi, Margaret M. Hayes, Rose L. Molina, Nicole H. Moraco, Anne Mosenthal, Michael Rosenblatt, Noa Talmor, Daniel P. Walsh, David N. Sontag, Jennifer P. Stevens

**Affiliations:** 1 Division of Pulmonary, Critical Care, and Sleep Medicine, Beth Israel Deaconess Medical Center, Boston, Massachusetts; 2 Harvard Medical School, Boston, Massachusetts; 3 Center for Healthcare Delivery Science, Beth Israel Deaconess Medical Center, Boston, Massachusetts; 4 Department of Anesthesia, Critical Care, and Pain Medicine, Beth Israel Deaconess Medical Center, Boston, Massachusetts; 5 Department of Obstetrics and Gynecology, Beth Israel Deaconess Medical Center, Boston, Massachusetts; 6 Division of General Surgery, Department of Surgery, Beth Israel Deaconess Medical Center, Boston, Massachusetts; 7 Division of Surgical Critical Care, Department of Surgery, Lahey Hospital and Medical Center, Burlington, Massachusetts; 8 Tufts University School of Medicine, Boston, Massachusetts; 9 Division of Critical Care, Beth Israel Deaconess Hospital–Plymouth, Plymouth, Massachusetts; 10 Office of the General Counsel, Beth Israel Lahey Health, Cambridge, Massachusetts; 11 Ethics Advisory Committee, Beth Israel Deaconess Medical Center, Boston, Massachusetts

## Abstract

**Importance:**

Crisis standards of care (CSOC) scores designed to allocate scarce resources during the COVID-19 pandemic could exacerbate racial disparities in health care.

**Objective:**

To analyze the association of a CSOC scoring system with resource prioritization and estimated excess mortality by race, ethnicity, and residence in a socially vulnerable area.

**Design, Setting, and Participants:**

This retrospective cohort analysis included adult patients in the intensive care unit during a regional COVID-19 surge from April 13 to May 22, 2020, at 6 hospitals in a health care network in greater Boston, Massachusetts. Participants were scored by acute severity of illness using the Sequential Organ Failure Assessment score and chronic severity of illness using comorbidity and life expectancy scores, and only participants with complete scores were included. The score was ordinal, with cutoff points suggested by the Massachusetts guidelines.

**Exposures:**

Race, ethnicity, Social Vulnerability Index.

**Main Outcomes and Measures:**

The primary outcome was proportion of patients in the lowest priority score category stratified by self-reported race. Secondary outcomes were discrimination and calibration of the score overall and by race, ethnicity, and neighborhood Social Vulnerability Index. Projected excess deaths were modeled by race, using the priority scoring system and a random lottery.

**Results:**

Of 608 patients in the intensive care unit during the study period, 498 had complete data and were included in the analysis; this population had a median (IQR) age of 67 (56-75) years, 191 (38.4%) female participants, 79 (15.9%) Black participants, and 225 patients (45.7%) with COVID-19. The area under the receiver operating characteristic curve for the priority score was 0.79 and was similar across racial groups. Black patients were more likely than others to be in the lowest priority group (12 [15.2%] vs 34 [8.1%]; *P* = .046). In an exploratory simulation model using the score for ventilator allocation, with only those in the highest priority group receiving ventilators, there were 43.9% excess deaths among Black patients (18 of 41 patients) and 28.6% (58 of 203 patients among all others (*P* = .05); when the highest and intermediate priority groups received ventilators, there were 4.9% (2 of 41 patients) excess deaths among Black patients and 3.0% (6 of 203) among all others (*P* = .53). A random lottery resulted in more excess deaths than the score.

**Conclusions and Relevance:**

In this study, a CSOC priority score resulted in lower prioritization of Black patients to receive scarce resources. A model using a random lottery resulted in more estimated excess deaths overall without improving equity by race. CSOC policies must be evaluated for their potential association with racial disparities in health care.

## Key Points

**Question:**

Is a crisis standards of care scoring system designed to allocate scarce resources in the COVID-19 pandemic associated with inequities in resource allocation by race?

**Findings:**

In this cohort study of 498 adults admitted to the intensive care unit and preemptively scored during a COVID-19 surge, nearly twice the proportion of Black patients were scored in the lowest priority group compared with all other patients, a significant difference.

**Meaning:**

These findings suggest that a scoring system designed to maximize lives and life-years saved in the setting of resource scarcity during the COVID-19 pandemic may result in racial inequities in prioritization.

## Teaser

This cohort study analyzes the association of a crisis standards of care scoring system with resource prioritization and estimated excess mortality by race, ethnicity, and residence in a socially vulnerable area.

## Introduction

During the COVID-19 pandemic, regional surges in the number of critically ill patients led to concerns that available critical care resources, including ventilators as well as staffed intensive care unit (ICU) beds, could be inadequate to meet patient needs.^1^ In response, crisis standards of care (CSOC) plans for allocation of resources were developed.^2^ Initial ethical frameworks for CSOC plans focused primarily on prioritizing the greatest number of lives saved and life-years saved.^3,4,5^ Policies to operationalize these frameworks most often included the Sequential Organ Failure Assessment (SOFA) score for estimating the likelihood of acute mortality and assessment of life expectancy or comorbidities for longer-term mortality prediction.^2,6,7^

Advocacy groups and ethicists, citing the known impact of structural racism on health outcomes before and during the COVID-19 pandemic,^8,9^ subsequently highlighted the need to include equity as a primary goal of CSOC triage scores.^2,10,11,12,13,14^ Scoring systems could exacerbate racial inequities in several ways. In some cases, bedside clinicians participate in scoring, and explicit or implicit biases could play a role in perpetuating disparities.^10^ Scores could have differential discrimination or calibration characteristics in different racial groups, which could result in worsening inequities.^15,16^ Finally, because poor health status is an outcome of structural racism, allocating resources first to patients who are less sick could increase already-present disparities; this is a potential problem even if scoring systems perfectly estimate the likelihood of mortality.^10,11,12,17^

A few studies have begun to look at the question of the equity impact of CSOC systems. One large retrospective study of patients with sepsis or acute respiratory failure prior to the COVID-19 pandemic found that 2 common scores of acute severity of illness were likely to perpetuate structural racism because of worse calibration of the score in Black patients.^16^ Another large retrospective study of ICU patients admitted prior to the COVID-19 pandemic also found that the SOFA score overestimated mortality among Black patients.^18^ Another study that looked at 3 priority groupings of critically ill patients in 2 hospitals in Florida during the pandemic found no difference in priority groupings between patients in different racial categories.^7^

The Massachusetts Department of Public Health published guidelines for the allocation of scarce resources on April 7, 2020, using the SOFA score as a proxy for likelihood of acute survival and a comorbidities score as a proxy for likelihood of long-term survival.^19^ After advocates raised concerns that the higher prevalence of comorbidities in underrepresented minority groups and people with disabilities would result in an inequitable distribution of resources,^13,20,21,22,23^ revised guidelines were published on April 20, 2020,^24^ using a clinician assessment of life expectancy to estimate likelihood of survival after hospitalization. During a regional surge in cases of COVID-19, our hospital network in Greater Boston preemptively scored patients to prepare for the imminent possibility of inadequate critical care resources, including ventilators and staffed ICU beds. With multiple efforts, including expansion of ICU beds and ventilators, resources remained adequate, so allocation by score did not occur. We retrospectively analyzed the scoring data to assess the proportion of people in each priority group and the capacity of the scores to estimate the likelihood of survival overall and by race, ethnicity, and the US Centers for Disease Control and Prevention Social Vulnerability Index (SVI). We also performed exploratory analyses by modeling scenarios in which ventilator capacity was inadequate and comparing mortality outcomes by race using the CSOC scoring system and a random allocation method.

## Methods

The institutional review board of the Beth Israel Deaconess Medical Center reviewed the study and deemed it exempt. Informed consent was not required, as this was a retrospective medical record review that was of minimal risk to patients. We used the Strengthening the Reporting of Observational Studies in Epidemiology (STROBE) guidelines checklist for cohort studies to structure our reporting of the study.

### Study Population

We performed a retrospective cohort analysis of all patients admitted to the ICUs of 6 Boston-area tertiary and community hospitals in the Beth Israel Lahey Hospital system (eTable 1 in the Supplement) for any reason between April 13 and May 22, 2020. Only those who had priority scores completed during their admission were included in the analyses.

### Data Collection

Beginning on April 13, 2020, ICU patients’ data were extracted from the electronic medical record by redeployed nurses. The nurses collected the variables of the SOFA score^25^ and ranked the presence and severity of comorbidities from the medical record using a 3-level score (Table 1). Demographic information (age, sex, race, ethnicity, and zip code) was also collected and recorded.

**Table 1.** Crisis Standards of Care Priority Scoring System

Principle	Specification	Point system			
		1 Point	2 Points	3 Points	4 Points
Save lives	Prognosis for short-term survival (SOFA score)	SOFA score <6	SOFA score 6-9	SOFA score 10-12	SOFA score >12
Save life-years^a^					
Apr 13-27, 2020	Prognosis for long-term survival of the immediate illness (medical assessment of comorbid conditions)	NA	Major comorbid conditions with substantial impact on long-term survival of immediate illness	NA	Severely life limiting conditions; death likely within 1 y
Apr 28 to May 22, 2020	Prognosis for continued survival (medical assessment of underlying conditions that severely limit life expectancy)	NA	Major underlying conditions that significantly limit near-term prognosis; death likely within 5 y	NA	Severely life-limiting conditions; death likely within 1 y

Abbreviations: NA, not applicable; SOFA, Sequential Organ Failure Assessment.

^a^ On April 28, 2020, institutions began to use an estimate of life expectancy instead of a measure of comorbidities in response to the Massachusetts revised guidelines. Attending physicians were asked to respond to the following questions: (1) “In your best clinical judgment based on the patient’s known underlying conditions that preceded this acute illness, do you think this patient is likely to survive more than 1 year? If answer is NO, then Score +4 points”; (2) “In your best clinical judgment based on the patient’s known underlying conditions that preceded this acute illness, do you think this patient is likely to survive more than 5 years? If answer is NO, then Score +2 points”; and (3) “If, in your best clinical judgment, you think the patient IS likely to survive more than 5 years, then Score 0 points.”

On April 28th, 2020, institutions began to use an estimate of life expectancy instead of comorbidities in response to the Massachusetts revised guidelines.^24^ Attending physicians were asked for an assessment of the likelihood that a patient would survive past 1 year or 5 years, based on their baseline health status at the time of ICU admission. A footnote in Table 1 includes the exact questions posed to attending physicians. Subsequently, discharge dates, vital status at discharge, and discharge destination were abstracted from the electronic medical record for this study.

### Study Variables

#### CSOC Components and Score

The CSOC prioritization scoring system was outlined by the commonwealth of Massachusetts for application in individual hospitals.^24^ It was an aggregate score based on points derived from a patient’s SOFA score and either their comorbidities or their estimated life expectancy. The SOFA score was converted into a 4-point scale based on the following: 1 point for SOFA score less than 6, 2 points for SOFA score 6 to 9, 3 points for SOFA score 10 to 12, and 4 points for SOFA score greater than 12 (Table 1). Comorbidities were based on a 3-level system: 0 points for no significant comorbidities; 2 points for major comorbid conditions with substantial impact on long-term survival; and 4 points for severely life-limiting conditions prior to the acute illness. Life expectancy was also a 3-level score: 0 points for death not likely in 5 years; 2 points for death likely within 5 years; and 4 points for death likely within 1 year (Table 1).

Point scores were summed to create a raw ordinal priority score ranging from 1 to 8. Priority scores were then categorized as highest, intermediate, or lowest priority, according to commonwealth recommendations.^24^ Highest priority included scores 1 to 2; intermediate included scores 3 to 5; and lowest included scores 6 to 8. If the CSOC decisions had been activated, patients in the highest priority group would have been the first to receive scarce critical care resources, followed by the intermediate and then lowest priority groups. While these were the 3 categories in the state recommendations, actual allocation cutoffs in the ordinal score would be based on the gap between supply and demand of resources at the time when the system needed to be implemented.

#### Demographic Characteristics

Demographic characteristics included in the analyses were age, sex, self-reported race, self-reported Hispanic ethnicity, and residence in socially vulnerable areas, using home zip codes and categorized by SVI.^26^ Both race and Hispanic ethnicity were reported by patients or their surrogates as part of the hospital admission registration process and recorded in the medical record. Each site used its own script as part of its usual registration process to elicit race and ethnicity at the time of admission, and the answers were noted in the medical record. We then abstracted race and ethnicity from the medical record for the study. Race was categorized as other for any self-reported race reported in the medical record that was not White, Black, or Asian. It was listed as unknown when no self-reported race was recorded. For ethnicity, patients or surrogates reported Hispanic ethnicity or not Hispanic ethnicity. If no answer was recorded in the medical record, this was listed as unknown. The SVI is a measure of vulnerability based on socioeconomic status, household composition, race, ethnicity, language, housing, and transportation. We defined socially vulnerable areas as those in the top quartile of the SVI.

#### Outcomes

The primary outcome was the proportion of patients in the lowest priority score group stratified by race. Secondary outcomes were discrimination and calibration of the score overall and by race, ethnicity, and neighborhood SVI. We also modeled projected excess deaths by race for patients receiving ventilation, using both the priority scoring system and a random lottery.

### Statistical Analysis

#### Descriptive Statistics

We presented continuous variables using median and IQR, and categorical variables using counts and proportions. We used overall χ^2^ tests for categorical variables and Kruskal-Wallis tests for continuous variables to compare patient characteristics, outcomes, and scores by individual racial groups, Black vs all other groups, and Black vs White. Secondary analyses compared patient characteristics, outcomes, and scores across ethnicity and residence in socially vulnerable areas.

#### CSOC Score Performance

We evaluated the discrimination of the priority score to estimate the likelihood of in-hospital mortality using the area under the receiver operating characteristic curve (AUROC), and the calibration using Hosmer-Lemeshow test of difference. We analyzed these for the entire population and stratified by racial group, ethnicity, and residence in socially vulnerable areas.

#### Priority Score vs Random Lottery Model in Patients Receiving Ventilation

We conducted exploratory scenario testing to evaluate simulated mortality outcomes overall and by race, using our CSOC score vs a random lottery in the subset of patients who were receiving ventilation. We postulated a scenario of scarcity requiring allocation of ventilators using 2 state-recommended cutoffs: score of 2 or less (ie, only highest priority category patients receive a ventilator; ventilator deficit of 140), and score of 5 or less (both highest priority and intermediate priority category patients receive a ventilator; ventilator deficit of 30.) We ran 10 000 trials in which individuals were randomly assigned to receive a ventilator (where 140 individuals did not receive a ventilator in the highest priority cutoff simulation and 30 individuals did not receive a ventilator in the highest and intermediate priority cutoff simulation.) Baseline deaths in this model were those who actually died in our cohort of patients receiving ventilation. Excess deaths were those patients who actually lived in our cohort but did not receive a ventilator in the model and therefore were assumed to have died in the scenario. We compared overall in-hospital mortality with the priority score and random lottery methods as well as mortality by race using each system.

All tests were 2-tailed; *P* < .05 was considered statistically significant. Statistical analyses were done using SAS version 9.4 (SAS Inc) and Stata SE version 16 (StataCorp).

## Results

### Cohort and Missing Data

During the study period, 608 unique patients were present in the ICUs of the 6 hospitals. Of these, 3 had only demographic data recorded, and an additional 107 had only demographic and SOFA score recorded; the 498 patients with complete data were included in the analyses (eFigure 1 in the Supplement). A comparison of patients with complete vs missing data revealed no significant difference for the baseline characteristics of age, sex, COVID-19 positivity, and residence in a socially vulnerable area (eTable 2 in the Supplement). There was no significant difference between the proportions of Black and White patients by complete vs missing data, and no difference between the proportions of Hispanic and non-Hispanic patients; there was a significant difference when all races and ethnicities including unknown and other were compared to each other, with a higher proportion of unknown race and ethnicity in the patients with missing score data (eTable 2 in the Supplement). Most outcomes were not significantly different for those with complete vs missing data; however, those with complete data had significantly more days receiving ventilation and lower likelihood of discharge home.

### Patient Characteristics

The median (IQR) age was 67 (56-75) years, and 191 (38.4%) were female patients (Table 2). Black race was self-reported by 79 patients (15.9%); White, by 298 (59.8%); Asian, by 11 (2.2%); and other, by 46 (9.2%); 64 (12.9%) did not have a reported race. Of participants, 55 (11.0%) reported Hispanic ethnicity, and 127 (25.5%) resided in socially vulnerable areas (eTables 3 and 4 in the Supplement).

**Table 2.** Patient Characteristics and Outcomes Overall and by Race

Characteristic	Patients, No. (%)						*P* value	
	Overall (N = 498)	Black (n = 79)	White (n = 298)	Asian (n = 11)	Other (n = 46)^a^	Unknown (n = 64)	Black vs White	Black vs all others^b^
Demographic and clinical characteristics								
Age, median (IQR), y	67 (56-75)	68 (59-75)	69 (57-76)	62 (59-72)	63 (52-73)	59 (50-69)	.76	.43
Female	191 (38.4)	26 (32.9)	117 (39.3)	4 (36.4)	15 (32.6)	29 (45.3)	.30	.28
Male	307 (61.6)	53 (67.1)	181 (60.7)	7 (63.6)	31 (67.4)	35 (54.7)		
COVID-19 positive	225 (45.7)	57 (72.2)	94 (31.5)	9 (81.8)	29 (63)	36 (56.3)	<.001	<.001
Outcomes								
Ventilated	244 (49.5)	41 (51.9)	120 (40.3)	7 (63.6)	26 (56.5)	50 (78.1)	.08	.64
Time on ventilator, median (IQR), d	10 (4-19)	15 (6-25)	8 (3-16)	10 (6-12)	15 (7-23)	14 (7-23)	.01	.10
LOS, median (IQR), d								
ICU	6 (3-17)	8 (3-21)	5 (3-11)	13 (8-18)	22 (25-31)	16 (8-26)	.004	.19
Hospital	13 (7-25)	13 (8-28)	10 (6-19)	18 (9-31)	17 (9-38)	22 (13-31)	.01	.26
Discharged home	165 (45.3)	19 (24.1)	108 (36.2)	3 (27.3)	19 (41.3)	16 (25)	.13	.13
In-hospital death	119 (23.9)	21 (26.6)	62 (20.8)	3 (27.3)	14 (30.4)	19 (29.7)	.27	.54

Abbreviations: ICU, intensive care unit; LOS, length of stay.

^a^ Other includes any race indicated by the patient or surrogate that was not Black, White, or Asian.

^b^ All others includes all other races as well as patients with unknown race.

Of all participants, 225 (45.7%) were COVID-19 positive (Table 2). As compared with White patients, Black patients had a higher prevalence of COVID-19 infection (94 [31.5%] vs 57 [72.2%]; *P* < .001) (Table 2). Hispanic patients had similar rates of COVID-19 infection as non-Hispanic patients (23 [41.8%] vs 165 [43.9%]; *P* = .84) (eTable 3 in the Supplement). Patients residing in socially vulnerable areas were more likely to be COVID-19 positive than others (79 [62.2%] vs 146 [39.4%]; *P* < .001) (eTable 4 in the Supplement).

Given the large difference in COVID-19 prevalence among Black vs other patients, we also examined characteristics of patients with COVID-19 and those without COVID-19 as separate subpopulations (eTables 5 and 6 in the Supplement). There was no significant difference in characteristics between Black and White patients within either the COVID-19–positive or COVID-19–negative subpopulations.

### Patient Outcomes

Overall, 244 patients (49.5%) received mechanical ventilation, with a median (IQR) of 10 (4-19) days of ventilation (Table 2). Median (IQR) ICU length of stay was 6 (3-17) days, with median (IQR) hospital length of stay of 13 (7-25) days. A total of 119 patients (23.9%) died during the hospitalization, and 165 (45.3%) were discharged home without hospice.

Some patient outcomes varied by race. Compared with White patients, Black patients had longer median (IQR) length of mechanical ventilation (8 [3-16] days vs 15 [6-25] days; *P* = .01), longer ICU length of stay (5 [3-11] days vs 8 [3-21] days; *P* = .004), and longer hospital length of stay (10 [6-19] days vs 13 [8-28] days; *P* = .01) (Table 2). There was no statistically significant difference between Black and White patients in likelihood of death or discharge home (death: 21 [26.6%] vs 62 [20.8%]; *P* = .27; discharged home: 19 [24.1%] vs 108 [36.2%]; *P* = .13). When separated into COVID-19 and non–COVID-19 subpopulations, the only statistically significant difference in outcomes between Black and White patients was median (IQR) ICU LOS in the non–COVID-19 population (7 [3-18] days vs 4 [3-7] days; *P* = .03) (eTables 5 and 6 in the Supplement).

There was no statistically significant difference in outcomes between Hispanic and non-Hispanic patients, including in-hospital mortality (14 [25.5%] vs 85 [22.6%]; *P* = .69) (eTable 3 in the Supplement). Patients from socially vulnerable areas had statistically similar outcomes for in-hospital death and discharge home; however, they were more likely to receive ventilation (76 [59.8%] vs 168 [45.3%]; *P* = .007), to receive ventilation longer (median [IQR], 15 [6-25] days vs 9 [4-17] days; *P* = .005), have longer median (IQR) ICU stays (12 [3-26] days vs 5 [3-14] days; *P* < .001), and longer median (IQR) hospital stays (19 [9-35] days vs 11 [6-22] days, *P* < .001) (eTable 4 in the Supplement).

### Priority Scores by Race, Ethnicity, and Socially Vulnerable Area

Black patients were more likely to be in the lowest priority group compared with other patients (12 [15.2%] vs 34 [8.1%]; *P* = .046, Table 3). This was our primary outcome of interest. None of the component scores of the priority score (SOFA, comorbidities, life expectancy scores) demonstrated a statistically significant difference between racial groups (Table 3). When we analyzed for differences across all priority groups, we did not find a significant difference by race (eTable 7 in the Supplement).

**Table 3.** Proportion of Patients in the Lowest Priority Score Group for Overall Priority Score and Component Scores, Black vs White and Black vs All Others

Groups	Patients, No. (%)			*P* value^a^	Patients, No. (%)		*P* value^a^
	Overall (n = 498)	Black (n = 79)	White (298)		Black (n = 79)	All others (n = 419)^b^	
**Priority group**							
Lowest priority group (most severe, scores 6-8)	46 (9.2)	12 (15.2)	25 (8.4)	.07	12 (15.2)	34 (8.1)	.046
Higher priority groups (less severe, scores 1-5)	452 (90.7)	67 (84.8)	273 (91.6)		67 (84.8)	385 (91.9)	
**SOFA group**							
Lowest priority SOFA group (most severe, group 4)	33 (6.6)	7 (8.9)	14 (4.7)	.15	7 (8.9)	26 (6.2)	.38
Higher priority SOFA groups (less severe, groups 1-3)	465 (93.4)	72 (91.1)	284 (95.3)		72 (91.1)	393 (93.8)	
**Comorbidity points^c^**							
Lowest priority comorbidity group (most severe, score 4)	19 (3.8)	3 (3.8)	14 (10.9)	.69	3 (8.6)	16 (8.2)	.99
Higher priority comorbidity groups (less severe, scores 0 or 2)	211 (91.7)	32 (91.4)	115 (89.2)		32 (91.4)	179 (91.8)	
**Life expectancy points^c^**							
Lowest priority life expectancy group (most severe, score 4)	43 (16.0)	9 (20.4)	26 (15.4)	.41	9 (20.4)	34 (15.2)	.38
Higher priority life expectancy groups (less severe, scores 0 or 2)	225 (84.0)	35 (79.6)	143 (84.6)		35 (79.6)	190 (84.8)	

Abbreviation: SOFA, Sequential Organ Failure Assessment.

^a^ The *P* values reflect the comparison of patients in the lowest priority grouping for the overall priority score as well as the component scores (ie, SOFA, life expectancy, comorbidity) compared with all other higher-priority score values. Overall χ^2^ test was used to test difference across groups. Fisher exact test was used for cell counts less than 5.

^b^ All others includes all other races as well as patients with unknown race.

^c^ On April 28, 2020, institutions began to use an estimate of life expectancy instead of a measure of comorbidities in response to the Massachusetts revised guidelines. A total of 230 patients had comorbidity scores; 268 had life expectancy scores. Column percentages for comorbidity points and life expectancy points were based on these totals.

The SOFA score component was significantly higher for Hispanic patients as compared with others; otherwise, there was no significant difference in the overall prioritization score or other components of the score by ethnicity (eTable 8 in the Supplement). Neither the overall nor component priority scores were statistically different by patients residing in socially vulnerable areas vs others (eTable 9 in the Supplement). Within the COVID-19 and non–COVID-19 subpopulations, there was no difference in priority group by race (eTables 10-13 in the Supplement).

### CSOC Score Performance

**Figure 1.**
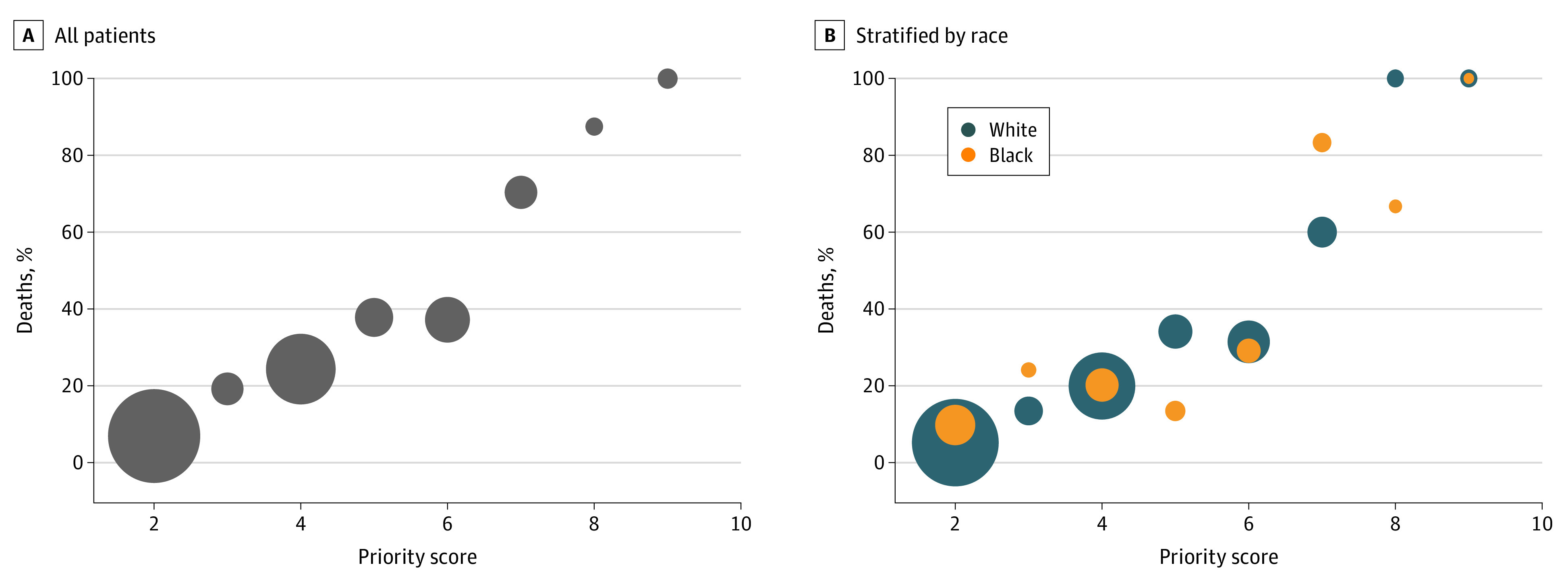
In-Hospital Mortality by Priority Score In-hospital mortality by priority score for all patients (A) and White and Black patients (B). The size of the bubble represents the total number of patients in each priority score group.

The scoring system had an AUROC of 0.79 and performed similarly across racial designations (Black, 0.76; White, 0.78; Asian, too few to calculate; other, 0.83; and unknown, 0.82) (Figure 1 and eFigure 2 and eTable 14 in the Supplement). It also had similar performance by ethnicity and socially vulnerable area (Hispanic ethnicity, 0.82; and non-Hispanic ethnicity, 0.79; socially vulnerable areas, 0.81; and non–socially vulnerable areas, 0.78) (eFigures 2-4 and eTable 14 in the Supplement). When separated into COVID-19 and non–COVID-19 sub-populations, AUROC was similar by race, other than an AUROC of 0.58 for Black patients with no COVID-19 (eTable 15 in the Supplement). This latter group consisted of only 22 patients.

For calibration, the Hosmer-Lemeshow test was not significant (*P* = .19) overall, with 0.43 for Black patients and 0.50 for White patients (Figure 1; eTable 14 in the Supplement). Additional tests of calibration across ethnicity and residence in a socially vulnerable area are presented in eFigures 3 and 4 and eTable 14 in the Supplement.

### Model of Outcomes With Priority Score vs Random Lottery

**Figure 2.**
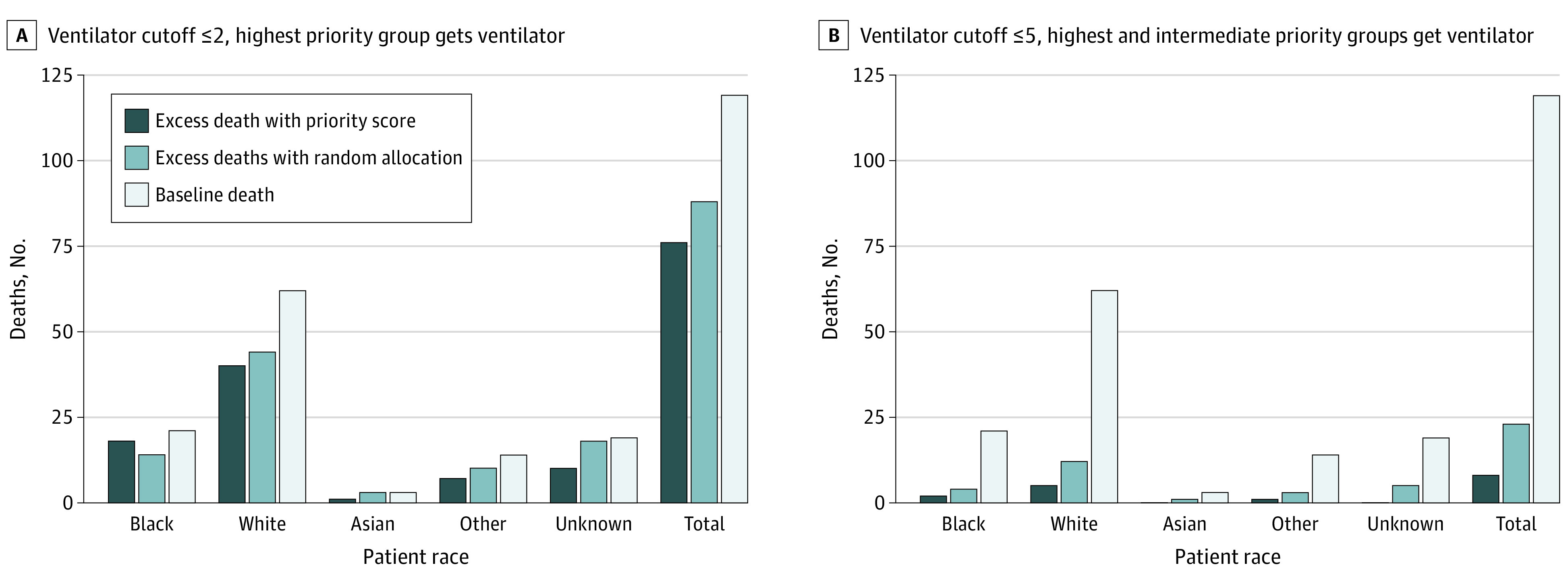
Comparison of Priority Score Allocation Strategy vs Random Lottery Allocation in Scenarios of Scarcity Baseline deaths defined as how many patients in our cohort died. Excess deaths defined as how many patients lived in our cohort but were estimated to have died in each scenario if we had to ration resources. For the random lottery allocation of ventilators at cutoffs of score of 2 or lower and 5 or lower, we ran 10 000 trials in which we randomly assigned individuals to receive a ventilator (where 140 individuals did not receive a ventilator at cutoff ≤2 and 30 individuals did not receive a ventilator at cutoff ≤5, including only patients who actually received ventilation in our cohort). Other race includes any race indicated by the patient or surrogate that was not Black, White, or Asian.

Figure 2 depicts an exploratory model of the number of estimated excess deaths overall and by racial group if a relative lack of mechanical ventilators required allocating ventilators to a priority score of 2 or less (highest priority group receives ventilators; Figure 2A) or priority score of 5 or less (highest and intermediate priority groups receive ventilators; Figure 2B) (eTables 16 and 17 in the Supplement). The model includes only the 244 patients who received ventilation.

With only those in the highest priority group receiving ventilators (score ≤2), there were 43.9% excess deaths among Black patients (18 of 41 patients) and 28.6% (58 of 203 patients) among all others (*P* = .05) (Figure 2 and eTable 16 in the Supplement). With the highest and intermediate priority groups receiving ventilators (score ≤5), excess deaths among Black patients were estimated to be 4.9% (2 of 41) vs 3.0% (6 of 203) for all others (*P* = .53) (Figure 2 and eTable 16 in the Supplement).

Providing ventilators to all patients in the highest priority score group (score ≤2) would result in 31.2% excess deaths (76 of 244 patients); using a random allocation system instead in this scenario would result in 35.9% excess deaths (88 patients) (*P* = .06) (Figure 2; eTable 17 in the Supplement). With cutoff of score 5 or lower (highest and intermediate priority groups get ventilators), there were 3.3% estimated excess deaths using the score (8 patients) and 9.6% using random allocation (23 patients) (*P* < .001) (Figure 2; eTable 17 in the Supplement). Estimated excess deaths were significantly lower using the priority score compared with random allocation for all possible cutoffs other than scores of 2 or less (eTable 17 in the Supplement). There was no consistent indication of whether the score or random allocation system resulted in lower excess deaths within any racial group (eTable 17 in the Supplement).

## Discussion

In our cohort of 498 ICU patients with and without COVID-19 during the pandemic, self-identified Black patients were significantly more likely to be in the lowest prioritization category to receive critical care resources compared with other patients. No significant difference in prioritization score was seen by Hispanic ethnicity or socially vulnerable area. The score had moderate discrimination and calibration performance in all groups.

In our exploratory simulation model of patients receiving ventilation, we found that the priority score resulted in statistically higher excess deaths for Black patients compared with all others for some cutoffs but not others. We also found that random allocation resulted in more overall excess deaths than the priority score, without a significant association with proportion of excess deaths within racial groups.

A combined acute and long-term severity of illness score has moderate discrimination for in-hospital death, suggesting the CSOC score identified many patients likely to die during the hospitalization. Black patients had higher rates of COVID-19 infection as their underlying illness and were admitted with higher levels of combined acute and chronic severity of illness. They therefore received lower prioritization for critical care resources. If higher severity of illness scores among Black patients reflect the impact of systemic racism on health,^10^ the CSOC system could perpetuate the impact of racism on health by deprioritizing patients with more severe illness. This is true even with a severity of illness score that is relatively good at estimating the likelihood of death among all racial groups. Although advocacy in Massachusetts focused on the comorbidities score as the potential source of perpetuated inequities, it was only the combined score, whether including comorbidities or life expectancy estimates, that had significantly worse prioritization outcomes for Black patients in our cohort.

In addition, a random lottery, an alternative system that has been suggested to mitigate disparities,^12,27^ resulted in higher modelled excess deaths overall, without an indication of benefit for any racial group. Consideration of alternative systems in which the social construct of race is considered for an equity weight or correction factor for racism, in addition to a severity of illness score, could potentially maintain some of the value of resource allocation by estimated effectiveness of the resources while also mitigating the impact of systemic racism.^12,28,29^ Although multiple studies point to the inadequacy of severity of illness scores for making individual patient care and resource allocation decisions,^30,31,32^ it may be that maintaining some use of these scores maximizes lives saved within and across racial groups.

### Limitations

Our study has several limitations. First, it is based on 1 health system in greater Boston. While the health system includes tertiary academic hospitals as well as community hospitals, it is not the primary safety-net hospital system for the region and does not represent a random sample of patients in the region. This could create a selection bias within or across racial groups of unclear direction. Second, our study sample size was based on the data collected in preparation for a potential scenario of insufficient resources. It is likely that it was not adequately powered to detect all true differences in score performance and mortality across racial categories. We saw a significant difference in the proportion of patients in the lowest priority category when looking at Black patients vs patients identifying as another racial group; however, we did not see a significant difference in any of the individual contributing scores, similar to findings in a previous study.^7^ We also found that the simulated model using the score showed significantly higher estimated excess deaths for Black patients with some cutoffs for allocation but not for all. A larger data set with more power would enable us to investigate whether there is a consistent finding of inequitable prioritization by race. Third, the data were collected in the context of a regional surge in patients and stretched human resources; quality control was not performed. While this may affect the accuracy of the data, it also reflects how data would be collected in a crisis situation, with the outcomes reflecting this context. Fourth, we had a high rate of missing data for race and some missing data for the scores. This weakens the conclusions we can draw from our data.^28^ Fifth, we were only able to assess in-hospital mortality, not longer-term mortality. Especially given that this CSOC policy explicitly aimed to maximize life-years saved, in-hospital mortality is not the ideal outcome to measure. Sixth, we assumed the ability to create a cutoff based on a static deficit of ICU resources; in practice, the need for and supply of ICU resources would be dynamic. A multistate model would potentially give a better estimation of the outcomes of these policies; however, we did not have a data set adequate for multistate modeling, and it is likely that a simplified static model would have to be used in practice as well. We believe the somewhat simplified static analysis we have presented nonetheless provides some insight into the potential association of the scoring system with prioritization of resources by race.

Additionally, our model of excess deaths in the subpopulation of patients who received ventilation assumes that any patient who did not receive a ventilator in our model who had received one in real life would die. While this is an assumption we made for the model, we believe it is reasonable in the context of this study. We collected the data because we were in a crisis ICU capacity surge within the pandemic, and it appeared we might need to implement the system. During this time, we had already maximally expanded our ICU bed and ventilator capacity. We were already triaging only the patients with the most severe illness for ICU admission; we were also intubating patients when they had failed other respiratory therapies, not intubating preemptively to avoid deterioration. In this context, it is a reasonable assumption that not receiving intubation would have a high probability of resulting in death.

## Conclusions

Our hospital system tested a CSOC scoring system including both acute and chronic severity of illness parameters in preparation for a potential deficit in critical care resources during the COVID-19 pandemic. Had this scoring system been actually used, it could have led to resources being disproportionately allocated away from Black patients due to a higher proportion of Black patients falling in the lowest priority group based on severity of illness scoring. A model simulating an alternative random allocation of resources resulted in higher mortality overall and did not improve equity between racial groups.

Antiracism and equity are explicit ethical principles within CSOC planning. Antiracist policies are not defined by the ethics or intentions underlying them but are defined as those that produce or sustain racial equity.^33^ Ongoing assessment of outcomes with different CSOC policies in real-world settings should drive the development and modification of CSOC policies to dismantle structural racism and maximize equitable outcomes for patients.

## Notes

Supplement. **eTable 1. **Description of Hospitals in Study **eTable 2. **Characteristics and Outcomes for Patients With Complete vs Missing Priority Score Data **eTable 3. **Patient Characteristics and Outcomes by Hispanic Ethnicity **eTable 4. **Patient Characteristics and Outcomes by Socially Vulnerable Area **eTable 5. **Patient Characteristics and Outcomes Overall and by Race Among Patients With COVID-19 **eTable 6. **Patient Characteristics and Outcomes Overall and by Race Among Patients With No COVID-19 **eTable 7. **Detailed Scores Overall and by Race **eTable 8. **Detailed Scores by Ethnicity **eTable 9. **Detailed Scores by Socially Vulnerable Area **eTable 10. **Proportion of Patients in the Lowest Priority Score Group for Overall Priority Score and Component Scores, Black vs White and Black vs All Others, Among Patients With COVID-19 **eTable 11. **Proportion of Patients in the Lowest Priority Score Group for Overall Priority Score and Component Scores, Black vs White and Black vs All Others, Among Patients With No COVID-19 **eTable 12. **Detailed Scores Overall and by Race Among Patients With COVID-19 **eTable 13. **Detailed Scores Overall and by Race Among Patients With No COVID-19 **eTable 14.** Priority Score Discrimination and Calibration for In-Hospital Mortality, Stratified by Race and Hispanic Ethnicity **eTable 15.** Priority Score Discrimination and Calibration for In-Hospital Mortality, Stratified by Race and COVID-19 Status **eTable 16. **Estimated Excess Deaths by Race in Modelled Scenarios of Ventilator Scarcity **eTable 17. **Estimated Excess In-Hospital Deaths by Priority Score vs Random Allocation **eFigure 1. **Study Cohort **eFigure 2. **AUROCs or In-Hospital Mortality for All Patients (A), Black Patients (B), White Patients (C), Hispanic Patients (D), Non-Hispanic Patients (E), Patients From Socially Vulnerable Areas (F), and Patients From Non–Socially Vulnerable Areas **eFigure 3. **In-Hospital Mortality by Priority Score for White vs Asian Patients (A), White vs Other Race (B), White vs Unknown Race (C), and Hispanic vs Non-Hispanic Patients (D) **eFigure 4. **In-Hospital Mortality by Priority Score for Patients From Socially Vulnerable Areas and Non–Socially Vulnerable Areas
