# Supplementary material for: Assessment of a Crisis Standards of Care Scoring System for Resource Prioritization and Estimated Excess Mortality by Race, Ethnicity, and Socially Vulnerable Area During a Regional Surge in COVID-19
Source: JAMA Netw Open. 2022 Mar 15;5(3):e221744. doi: 10.1001/jamanetworkopen.2022.1744 (PMC8924715; doi:10.1001/jamanetworkopen.2022.1744)
Supplement: Supplement. — eTable 1. Description of Hospitals in Study eTable 2. Characteristics and Outcomes for Patients With Complete vs Missing Priority Score Data eTable 3. Patient Characteristics and Outcomes by Hispanic Ethnicity eTable 4. Patient Characteristics and Outcomes by Socially Vulnerable Area eTable 5. Patient Characteristics and Outcomes Overall and by Race Among Patients With COVID-19 eTable 6. Patient Characteristics and Outcomes Overall and by Race Among Patients With No COVID-19 eTable 7. Detailed Scores Overall and by Race eTable 8. Detailed Scores by Ethnicity eTable 9. Detailed Scores by Socially Vulnerable Area eTable 10. Proportion of Patients in the Lowest Priority Score Group for Overall Priority Score and Component Scores, Black vs White and Black vs All Others, Among Patients With COVID-19 eTable 11. Proportion of Patients in the Lowest Priority Score Group for Overall Priority Score and Component Scores, Black vs White and Black vs All Others, Among Patients With No COVID-19 eTable 12. Detailed Scores Overall and by Race Among Patients With COVID-19 eTable 13. Detailed Scores Overall and by Race Among Patients With No COVID-19 eTable 14. Priority Score Discrimination and Calibration for In-Hospital Mortality, Stratified by Race and Hispanic Ethnicity eTable 15. Priority Score Discrimination and Calibration for In-Hospital Mortality, Stratified by Race and COVID-19 Status eTable 16. Estimated Excess Deaths by Race in Modelled Scenarios of Ventilator Scarcity eTable 17. Estimated Excess In-Hospital Deaths by Priority Score vs Random Allocation eFigure 1. Study Cohort eFigure 2. AUROCs or In-Hospital Mortality for All Patients (A), Black Patients (B), White Patients (C), Hispanic Patients (D), Non-Hispanic Patients (E), Patients From Socially Vulnerable Areas (F), and Patients From Non–Socially Vulnerable Areas eFigure 3. In-Hospital Mortality by Priority Score for White vs Asian Patients (A), White vs Other Race (B), White vs Unknown Race (C), and Hispanic vs No [file jamanetwopen-e221744-s001.pdf]

Supplemental Online Content

Riviello ED, Dechen T, O'Donoghue AL, et al. Assessment of a crisis standards of care scoring system for resource prioritization and estimated excess mortality by race, ethnicity, and socially vulnerable area during a regional surge in COVID-19. *JAMA Netw Open.* 2022;5(3):e221744. doi:10.1001/jamanetworkopen.2022.1744

eTable 1. Description of Hospitals in Study

eTable 2. Characteristics and Outcomes for Patients With Complete vs Missing Priority Score Data

eTable 3. Patient Characteristics and Outcomes by Hispanic Ethnicity

eTable 4. Patient Characteristics and Outcomes by Socially Vulnerable Area

eTable 5. Patient Characteristics and Outcomes Overall and by Race Among Patients With COVID-19

eTable 6. Patient Characteristics and Outcomes Overall and by Race Among Patients With No COVID-19

eTable 7. Detailed Scores Overall and by Race

eTable 8. Detailed Scores by Ethnicity

eTable 9. Detailed Scores by Socially Vulnerable Area

eTable 10. Proportion of Patients in the Lowest Priority Score Group for Overall Priority Score and Component Scores, Black vs White and Black vs All Others, Among Patients With COVID-19

eTable 11. Proportion of Patients in the Lowest Priority Score Group for Overall Priority Score and Component Scores, Black vs White and Black vs All Others, Among Patients With No COVID-19

eTable 12. Detailed Scores Overall and by Race Among Patients With COVID-19

eTable 13. Detailed Scores Overall and by Race Among Patients With No COVID-19

eTable 14. Priority Score Discrimination and Calibration for In-Hospital Mortality, Stratified by Race and Hispanic Ethnicity

eTable 15. Priority Score Discrimination and Calibration for In-Hospital Mortality, Stratified by Race and COVID-19 Status

eTable 16. Estimated Excess Deaths by Race in Modelled Scenarios of Ventilator Scarcity

eTable 17. Estimated Excess In-Hospital Deaths by Priority Score vs Random Allocation

eFigure 1. Study Cohort

eFigure 2. AUROCs or In-Hospital Mortality for All Patients (A), Black Patients (B), White Patients (C), Hispanic Patients (D), Non-Hispanic Patients (E), Patients From Socially Vulnerable Areas (F), and Patients From Non–Socially Vulnerable Areas

eFigure 3. In-Hospital Mortality by Priority Score for White vs Asian Patients (A), White vs Other Race (B), White vs Unknown Race (C), and Hispanic vs Non-Hispanic Patients (D)

eFigure 4. In-Hospital Mortality by Priority Score for Patients From Socially Vulnerable Areas and Non–Socially Vulnerable Areas

© 2022 Riviello ED et al. *JAMA Network Open.*

This supplemental material has been provided by the authors to give readers additional information about their work.

© 2022 Riviello ED et al. *JAMA Network Open.*

eTable 1. Description of Hospitals in Study

Hospital	Type	Staffed Beds
Beth Israel Deaconess Medical Center - Boston	Tertiary	719
Lahey Hospital and Medical Center - Burlington	Tertiary	345
Beth Israel Deaconess Hospital - Plymouth	Community	164
Anna Jaques Hospital - Newburyport	Community	140
Beth Israel Deaconess Hospital - Milton	Community	102
Beth Israel Deaconess Hospital - Needham	Community	58

© 2022 Riviello ED et al. *JAMA Network Open.*

eTable 2. Characteristics and Outcomes for Patients With Complete vs Missing Priority Score Data

	Total (n=608)	Complete (n=498)	Missing[a] (n=110)	p-value[b]		
**Characteristics**						
Age, median (IQR)	66 (56-74)	67 (56-74)	64 (53-71)	0.06		
Female, n (%)	235 (38.8)	191 (38.4)	44 (40.7)	0.64		
					p-value (Black vs White)	p-value (Black vs all others[c])
Race, n (%)				0.03	0.23	0.06
Black	89 (14.6)	79 (15.9)	10 (9.1)			
White	356 (58.6)	298 (59.8)	58 (52.7)			
Asian	16 (2.6)	11 (2.2)	5 (4.6)			
other	61 (10.0)	46 (9.2)	15 (13.6)			
unknown	86 (14.1)	64 (12.9)	22 (20.0)			
Ethnicity, n (%)					p-value (Hispanic vs non-Hispanic)	p-value (Hispanic vs all others[d])
Hispanic	72 (11.8)	55 (11.0)	17 (15.5)	0.03	0.09	0.19
non-Hispanic	446 (73.4)	376 (75.5)	70 (63.6)			
unknown	90 (14.8)	67 (13.5)	23 (20.9)			
COVID-19 Positive	266 (44.9)	225 (45.7)	41 (41.0)	0.76		
Socially vulnerable area[e]	157 (25.8)	127 (25.5)	30 (27.3)	0.70		
**Outcomes**						
Ventilated, n (%)	293 (49.1)	244 (49.5)	49 (47.1)	0.65		
Days on ventilator, median (IQR)	9.5 (4-18)	10 (4-19)	6 (2-15)	0.01		
ICU LOS,[f] median (IQR)	6 (3-17)	6 (3-17)	5 (3-13)	0.12		
Hospital LOS,[f] median (IQR)	13 (7-24)	13 (7-25)	13 (7-21)	0.78		
Discharged home, n (%)	215 (47.8)	165 (45.3)	50 (58.1)	0.03		
Dead, n (%)	143 (23.5)	119 (23.9)	24 (21.8)	0.64		

[a] *Patients with missing data for all or components of the priority score.*
[b] *Test of difference across groups (overall Chi-square for categorical variables and Kruskal-Wallis for continuous variables.)*
[c] *All others here includes White, Asian, other and unknown races.*
[d] *All others here includes non-Hispanic and unknown ethnicities.*
[e] *CDC's social vulnerability index (SVI) is a measure of vulnerability based on socioeconomic status, household composition, race, ethnicity, language, housing and transportation based on a patient's zip code. We defined socially vulnerable areas (SVAs) as zip codes in the top quartile.*
[f] *Length of stay in days*

© 2022 Riviello ED et al. *JAMA Network Open.*

eTable 3. Patient Characteristics and Outcomes by Hispanic Ethnicity

		Ethnicity				
	Overall (n=498)	Hispanic (55)	non-Hispanic (n=376)	unknown (n=67)	p-value Hispanic vs non-Hispanic	p-value Hispanic vs all others[b]
**Characteristics**						
Age, median (IQR)	67 (56-75)	65 (54-71)	69 (59-76)	58 (49-68)	0.09	0.02
Female, n (%)	191 (38.4)	22 (40)	137 (36.4)	32 (47.8)	0.61	0.79
COVID-19 Positive, n (%)	225 (45.7)	23 (41.8)	165 (43.9)	37 (55.2)	0.84	0.72
**Outcomes**						
Ventilated, n (%)	244 (49.5)	27 (49.1)	167 (44.4)	50 (74.6)	0.48	0.93
Days on ventilator, median (IQR)	10 (4-19)	13 (3-29)	9 (4-17)	14 (7-23)	0.36	0.40
ICU LOS,[a] median (IQR)	6 (3-17)	8 (3-25)	5 (3-14)	15 (7-26)	0.56	0.13
Hospital LOS,[a] median (IQR)	13 (7-25)	16 (9-38)	11 (6-22)	21 (10-31)	0.13	0.04
Discharged home, n (%)	165 (45.3)	16 (29.1)	132 (35.1)	17 (25.4)	0.45	0.77
In-hospital death, n (%)	119 (23.9)	14 (25.5)	85 (22.6)	20 (29.9)	0.69	0.56

[a]*LOS is length of stay in days*
[b]*All others includes non-Hispanic and unknown*

© 2022 Riviello ED et al. *JAMA Network Open.*

eTable 4. Patient Characteristics and Outcomes by Socially Vulnerable Area (SVA)

		**Socially vulnerable area**		
	Overall (n=498)	SVA [a] (n=127)	non-SVA [a] (n=371)	p-value[b]
**Characteristics**				
Age, median (IQR)	67 (56-75)	67 (54-73)	67 (56-75)	0.23
Female, n (%)	191 (38.4)	55 (43.3)	136 (36.7)	0.18
COVID-19 Positive, n (%)	225 (45.7)	79 (62.2)	146 (39.4)	0.0001
**Outcomes**				
Ventilated, n (%)	244 (49.5)	76 (59.8)	168 (45.3)	0.0068
Days on ventilator, median (IQR)	10 (4-19)	15 (6-25)	9 (4-17)	0.0054
ICU LOS,[c] median (IQR)	6 (3-17)	12 (3-26)	5 (3-14)	0.0008
Hospital LOS,[c] median (IQR)	13 (7-25)	19 (9-35)	11 (6-22)	<.0001
Discharged home, n (%)	165 (45.3)	36 (28.3)	129 (34.8)	0.39
In-hospital death, n (%)	119 (23.9)	32 (25.2)	87 (23.5)	0.69

[a] CDC's social vulnerability index (SVI) is a measure of vulnerability based on socioeconomic status, household composition, race, ethnicity, language, housing and transportation based on a patient's zip code. We defined socially vulnerable areas (SVAs) as zip codes in the top quartile.
[b]Test of difference across all groups (overall Chi-square for categorical variables and Kruskal-Wallis for continuous variables)
[c]LOS is length of stay in days

© 2022 Riviello ED et al. *JAMA Network Open.*

eTable 5. Patient Characteristics and Outcomes Overall and by Race Among Patients With COVID-19

		Race						
	Overall (n=225)	Black (n=57)	White (n=94)	Asian (n=9)	other (n=29)	unknown (n=36)	p-value Black vs White	p-value Black vs all others[b]
**Characteristics**								
Age, median (IQR)	67 (58-74)	68 (59-77)	68 (61-76)	66 (59-72)	63 (54-71)	58 (47-67)	0.93	0.15
Female, n (%)	84 (37.3)	18 (31.6)	39 (41.5)	3 (33.3)	9 (31.0)	15 (41.7)	0.22	0.29
**Outcomes**								
Ventilated, n (%)	150 (66.9)	34 (59.6)	54 (58.1)	6 (66.7)	22 (75.9)	34 (94.4)	0.84	0.17
Days on ventilator, median (IQR)	15 (7-24)	16 (6-25)	14 (6-23)	11 (8-13)	16 (7-35)	16 (12-28)	0.85	0.67
ICU LOS,[a] median (IQR)	13 (5-25)	8 (3-21)	9 (4-21)	13 (8-14)	16 (8-35)	22 (13-31)	0.78	0.09
Hospital LOS,[a] median (IQR)	18 (9-33)	16 (9-29)	16 (8-30)	16 (9-21)	23 (12-29)	25 (19-42)	0.85	0.19
Discharged home, n (%)	83 (36.9)	9 (25.7)	8 (15.4)	3 (50.0)	10 (58.8)	9 (36.0)	0.23	0.63
In-hospital death, n (%)	39 (28.9)	18 (31.6)	39 (41.5)	3 (33.3)	12 (41.4)	11 (30.6)	0.22	0.33

[a] *LOS is length of stay in days.*
[b] *All others includes all other races, as well as unknown.*

© 2022 Riviello ED et al. *JAMA Network Open.*

eTable 6. Patient Characteristics and Outcomes Overall and by Race Among Patients With No COVID-19

non-COVID ONLY		Race						
	Overall (n=273)	Black (n=22)	White (n=204)	Asian (n=2)	other (n=17)	unknown (n=28)	p-value Black vs White	p-value Black vs all others[b]
**Characteristics**								
Age, median (IQR)	67 (54-75)	66 (53-73)	69 (56-77)	53 (43-62)	66 (43-73)	59 (52-72)	0.58	0.64
Female, n (%)	116 (60.8)	8 (36.4)	78 (38.2)	1 (50.0)	6 (35.3)	14 (50.0)	0.86	0.77
**Outcomes**								
Ventilated, n (%)	94 (34.9)	7 (31.8)	66 (33.0)	1(50.0)	4 (23.5)	16 (57.1)	0.91	0.74
Days on ventilator, median (IQR)	6 (2-12)	8 (5-21)	5 (2-10)	4 (4-4)	10 (3-16)	7 (3-15)	0.08	0.19
ICU LOS,[a] median (IQR)	4 (3-9)	7 (3-18)	4 (3-7)	15 (11-19)	5 (2-19)	16 (4-17)	0.03	0.08
Hospital LOS,[a] median (IQR)	10 (5-18)	11 (8-24)	9 (5-16)	37 (20-55)	12 (7-35)	16 (8-26)	0.11	0.14
Discharged home, n (%)	126 (55.0)	10 (55.6)	100 (56.8)	0	9 (69.2)	7 (35.0)	0.92	0.96
In-hospital death, n (%)	36 (13.2)	3 (13.6)	23 (11.3)	0	2(11.8)	8 (28.6)	0.74	0.94

[a] *LOS is length of stay in days.*
[b] *All others includes all other races, as well as unknown.*

© 2022 Riviello ED et al. *JAMA Network Open.*

eTable 7. Detailed Scores Overall and by Race

		Race					p-value[a] Black vs White	p-value[a] Black vs all others
	Overall (n=498)	Black (n=79)	White (n=298)	Asian (n=11)	other (n=46)	unknown (n=64)		
Priority Group,[b] n (%)							0.19	0.13
High	299 (60.0)	43 (54.4)	179 (60.1)	9 (81.8)	27 (58.7)	41 (64.1)		
Mid	153 (30.7)	24 (30.4)	94 (31.5)	2 (18.2)	12 (26.1)	21 (32.8)		
Low	46 (9.3)	12 (15.2)	25 (8.4)	0 (0)	7 (15.2)	2 (3.1)		
SOFA group,[c] n (%)							0.06	0.10
1- Least severe	358 (71.9)	49 (62)	224 (75.2)	9 (81.8)	31 (67.4)	45 (70.3)		
2	67 (13.4)	12 (15.2)	40 (13.4)	2 (18.2)	5 (10.9)	8 (12.5)		
3	40 (8.0)	11 (13.9)	20 (6.7)	0 (0)	3 (6.5)	6 (9.4)		
4- Most severe	33 (6.6)	7 (8.9)	14 (4.7)	0 (0)	7 (15.2)	5 (7.8)		
Comorbidity points,[d] n (%)							0.40	0.25
0-Least severe	133 (57.8)	16 (20.3)	72 (24.2)	5 (45.5)	14 (30.4)	26 (40.6)		
2	78 (33.9)	16 (20.3)	43 (14.4)	3 (27.3)	7 (15.2)	9 (14.1)		
4-Most severe	19 (8.3)	3 (3.8)	14 (4.7)	0 (0)	0 (0)	2 (3.1)		
Life expectancy points,[d] n (%)							0.72	0.68
0-Least severe	130 (48.5)	20 (25.3)	81 (27.2)	2 (18.2)	11 (23.9)	16 (25)		
2	95 (35.5)	15 (19)	62 (20.8)	1 (9.1)	8 (17.4)	9 (14.1)		
4-Most severe	43 (16.0)	9 (11.4)	26 (8.7)	0 (0)	6 (13)	2(3.1)		

[a]*Test of difference across groups (overall Chi-square)*

[b]*High priority score (0-2), mid priority score (3-5) and low priority score (6-8). Patients in the high priority group have the lowest severity of illness and are most likely to get critical care resources in periods of scarcity.*

[c]*1 point for acute SOFA score less than 6, 2 points for SOFA score between 6-9, 3 points for SOFA score 10-12, and 4 points for SOFA score greater than 12.*

[d] *On April 28th, 2020, institutions began to use an estimate of life expectancy instead of a measure of co-morbidities in response to the Massachusetts revised guidelines. Total patients with comorbidity scores are n=230; total patients with life expectancy scores are n=268. Column percentages for comorbidity points and life expectancy points were based on these totals.*

© 2022 Riviello ED et al. *JAMA Network Open.*

eTable 8. Detailed Scores by Ethnicity

		Ethnicity				
	Overall (n=498)	Hispanic (55)	non-Hispanic (n=376)	unknown (n=67)	p-value[a] Hispanic vs non-Hispanic	p-value[a] Hispanic vs all others
Priority Group,[b] n (%)					0.67	0.68
High	299 (60.0)	30 (54.5)	228 (60.6)	41 (61.2)		
Mid	153 (30.7)	19 (34.5)	110 (29.3)	24 (35.8)		
Low	46 (9.3)	6 (10.9)	38 (10.1)	2 (3)		
SOFA group,[c] n (%)					0.39	0.04
1- Least severe	358 (71.9)	32 (58.2)	278 (73.9)	48 (71.6)		
2	67 (13.4)	12 (21.8)	48 (12.8)	7 (10.4)		
3	40 (8.0)	4 (7.3)	28 (7.4)	8 (11.9)		
4- Most severe	33 (6.6)	7 (12.7)	22 (5.9)	4 (6)		
Comorbidity points,[d] n (%)					0.13	0.19
0- Least severe	133 (57.8)	10 (18.2)	98 (26.1)	25 (37.3)		
2	78 (33.9)	10 (18.2)	57 (15.2)	11 (16.4)		
4-Most severe	19 (8.3)	4 (7.3)	13 (3.5)	2 (3)		
Life expectancy points,[d] n (%)					0.58	0.53
0-Least severe	130 (48.5)	16 (29.1)	98 (26.1)	16 (23.9)		
2	95 (35.5)	12 (21.8)	73 (19.4)	10 (14.9)		
4-Most severe	43 (16.0)	3 (5.5)	37 (9.8)	3 (4.5)		

[a]*Test of difference across groups (overall Chi-square)*

[b]*High priority score (0-2), mid priority score (3-5) and low priority score (6-8). Patients in the high priority group have the lowest severity of illness and are most likely to get critical care resources in periods of scarcity.*

[c]*1 point for acute SOFA score less than 6, 2 points for SOFA score between 6-9, 3 points for SOFA score 10-12, and 4 points for SOFA score greater than 12.*

[d] *On April 28th, 2020, institutions began to use an estimate of life expectancy instead of a measure of co-morbidities in response to the Massachusetts revised guidelines. Total patients with comorbidity scores are n=230; total patients with life expectancy scores are n=268. Column percentages for comorbidity points and life expectancy points were based on these totals.*

© 2022 Riviello ED et al. *JAMA Network Open.*

eTable 9. Detailed Scores by Socially Vulnerable Area

		Socially vulnerable areas (SVA)[a]		
	Overall (n=498)	SVA (n=127)	non-SVA (n=371)	p-value[b]
Priority Group,[c] n(%)				0.31
High	299 (60.0)	73 (57.5)	226 (60.9)	
Mid	153 (30.7)	38 (29.9)	115 (31)	
Low	46 (9.3)	16 (12.6)	30 (8.1)	
SOFA group,[d] n(%)				0.16
1- Least severe	358 (71.9)	49 (62)	224 (75.2)	
2	67 (13.4)	12 (15.2)	40 (13.4)	
3	40 (8.0)	11 (13.9)	20 (6.7)	
4- Most severe	33 (6.6)	7 (8.9)	14 (4.7)	
Comorbidity points[e], n(%)				0.15
0- Least severe	133 (57.8)	32 (25.2)	101 (27.2)	
2	78 (33.9)	20 (15.7)	58 (15.6)	
4-Most severe	19 (8.3)	1 (0.8)	18 (4.9)	
Life expectancy points[e], n(%)				0.3
0-Least severe	130 (48.5)	34 (26.8)	96 (25.9)	
2	95 (35.5)	24 (18.9)	71 (19.1)	
4-Most severe	43 (16.0)	16 (12.6)	27 (7.3)	

[a]CDC's social vulnerability index (SVI) is a measure of vulnerability based on socioeconomic status, household composition, race, ethnicity, language, housing and transportation based on a patient's zip code. We defined socially vulnerable areas (SVAs) as zip codes in the top quartile.
[b]Test of difference across groups (overall Chi-square)
[c] High priority score (0-2), mid priority score (3-5) and low priority score (6-8). Patients in the high priority group have the lowest severity of illness and are most likely to get critical care resources in periods of scarcity.
[d] 1 point for acute SOFA score less than 6, 2 points for SOFA score between 6-9, 3 points for SOFA score 10-12, and 4 points for SOFA score greater than 12.
[e] On April 28th, 2020, institutions began to use an estimate of life expectancy instead of a measure of co-morbidities in response to the Massachusetts revised guidelines. Total patients with comorbidity scores are n=230; total patients with life expectancy scores are n=268. Column percentages for comorbidity points and life expectancy points were based on these totals.

© 2022 Riviello ED et al. *JAMA Network Open.*

eTable 10. **Proportion of Patients in the Lowest Priority Score Group for Overall Priority Score and Component Scores, Black vs White and Black vs All Others, Among Patients With COVID-19**

	Overall (n=225)	Black (n=57)	White (94)	p-value[a]		Black (n=57)	all others[b] (n=168)	p-value[a]
**Priority group, n (%)**								
Lowest priority group (most severe, scores 6-8)	33 (14.7)	11 (19.3)	15 (15.9)	0.59		11 19.3)	22 (13.1)	0.25
Higher priority groups (less severe, scores 1-5)	192 (85.3)	46 (80.7)	79 (84.0)			46 (80.7)	146 (80.7)	
**SOFA group, n (%)**								
Lowest priority SOFA group (most severe, group 4)	25 (11.1)	7 (12.3)	8 (8.5)	0.45		7 (12.3)	18 (10.7)	0.74
Higher priority SOFA groups (less severe, groups 1-3)	200 (88.9)	50 (87.7)	86 (91.5)			50 (87.7)	150 (89.3)	
**Co-morbidity points[c], n (%)**								
Lowest priority co-morbidity group (most severe, score 4)	6 (6.3)	2 (8.0)	4 (10.5)	0.73		2 (8.0)	4 (5.6)	0.67
Higher priority co-morbidity groups (less severe, scores 0 or 2)	90 (93.8)	23 (92.0)	34 (89.5)			23 (92.0)	67 (94.4)	
**Life expectancy points[c], n (%)**								
Lowest priority life expectancy group (most severe, score 4)	16 (28.6)	8 (25.0)	16 (28.6)	0.72		8 (25.0)	23 (23.7)	0.88
Higher priority life expectancy groups (less severe, scores 0 or 2)	40 (71.4)	24 (75.0)	40 (71.4)			24 (75.0)	74 (76.3)	

[a] *The p values reflect the comparison of patients in the lowest priority grouping for the overall priority score as well as the component scores (SOFA, life expectancy, co-morbidity), as compared with all other higher-priority score values. Overall Chi-square was used to test difference across groups. Fishers Exact was used for cell counts less than 5.*

[b] *All others includes all other races, as well as unknown.*

[c] *On April 28th, 2020, institutions began to use an estimate of life expectancy instead of a measure of comorbidities in response to the Massachusetts revised guidelines. Total patients with comorbidity scores are n=230; total patients with life expectancy scores are n=268. Column percentages for comorbidity points and life expectancy points were based on these totals.*

© 2022 Riviello ED et al. *JAMA Network Open.*

eTable 11. Proportion of Patients in the Lowest Priority Score Group for Overall Priority Score and Component Scores, Black vs White and Black vs All Others, Among Patients With No COVID-19

**non-COVID ONLY**	Overall (n=273)	Black (n=22)	White (204)	p-value[a]		Black (n=22)	all others[b] (n=251)	p-value[a]
**Priority group, n (%)**								
Lowest priority group (most severe, scores 6-8)	13 (4.8)	1 (4.6)	10 (4.9)	0.94		1 (4.6)	12 (4.8)	0.96
Higher priority groups (less severe, scores 1-5)	260 (95.2)	21 (95.4)	194 (95.1)			21 (95.4)	239 (95.2)	
**SOFA group, n (%)**								
Lowest priority SOFA group (most severe, group 4)	8 (2.9)	0	6 (2.9)			0	8 (3.2)	0.39
Higher priority SOFA groups (less severe, groups 1-3)	265 (97.1)	22 (100)	198 (97.1)			22 (100)	243 (96.8)	
**Co-morbidity points[c], n (%)**								
Lowest priority co-morbidity group (most severe, score 4)	13 (9.7)	1(10.0)	10 (11.0)	0.96		1(10.0)	12 (9.7)	0.97
Higher priority co-morbidity groups (less severe, scores 0 or 2)	121 (90.3)	9 (90.0)	81 (89.0)			9 (90.0)	112 (90.3)	
**Life expectancy points[c], n (%)**								
Lowest priority life expectancy group (most severe, score 4)	12 (8.6)	1 (8.3)	10 (8.9)	0.95		1 (8.3)	11 (8.7)	0.97
Higher priority life expectancy groups (less severe, scores 0 or 2)	127 (91.4)	11 (91.7)	103 (91.2)			11 (91.7)	116 (91.3)	

[a] *The p values reflect the comparison of patients in the lowest priority grouping for the overall priority score as well as the component scores (SOFA, life expectancy, co-morbidity), as compared with all other higher-priority score values. Overall Chi-square was used to test difference across groups. Fishers Exact was used for cell counts less than 5.*

[b] *All others includes all other races, as well as unknown.*

[c] *On April 28th, 2020, institutions began to use an estimate of life expectancy instead of a measure of comorbidities in response to the Massachusetts revised guidelines. Total patients with comorbidity scores are n=230; total patients with life expectancy scores are n=268. Column percentages for comorbidity points and life expectancy points were based on these totals.*

© 2022 Riviello ED et al. *JAMA Network Open.*

eTable 12. Detailed Scores Overall and by Race Among Patients With COVID-19

	Overall (n=225)	Race: Black (n=57)	White (n=94)	Asian (n=9)	other (n=29)	unknown (n=36)	p-value[a] Black vs White	p-value[a] Black vs all others
Priority Group,[b] n (%)								
Low	33 (14.7)	11 (19.3)	15 (16)	0 (0)	6 (20.7)	1 (2.8)	0.74	0.51
Mid	75 (33.3)	28 (49.1)	44 (46.8)	7 (77.8)	17 (58.6)	21 (58.3)		
High	117 (52.0)	18 (31.6)	35 (37.2)	2 (22.2)	6 (20.7)	14 (38.9)		
SOFA group,[c] n (%)								
1- Least severe	137 (60.9)	32 (56.1)	59 (62.8)	8 (88.9)	17 (58.6)	21 (58.3)	0.7	0.7
2	33 (14.7)	8 (14)	15 (16)	1 (11.1)	3 (10.3)	6 (16.7)		
3	30 (13.3)	10 (17.5)	12 (12.8)	0 (0)	2 (6.9)	6 (16.7)		
4- Most severe	25 (11.1)	7 (12.3)	8 (8.5)	0 (0)	7 (24.1)	3 (8.3)		
Comorbidity points,[d] n (%)								
0-Least severe	53 (55.2)	11 (19.3)	22 (23.4)	4 (44.4)	5 (17.2)	11 (30.6)	0.42	0.43
2	37 (38.5)	12 (21.1)	12 (12.8)	3 (33.3)	5 (17.2)	5 (13.9)		
4-Most severe	6 (6.3)	2 (3.5)	4 (4.3)	0 (0)	0 (0)	0 (0)		
Life expectancy points,[d] n (%)								
0-Least severe	52 (40.3)	12 (21.1)	15 (16)	1 (11.1)	11 (37.9)	13 (36.1)	0.57	0.93
2	46 (35.7)	12 (21.1)	25 (26.6)	1 (11.1)	3 (10.3)	5 (13.9)		
4-Most severe	31 (24.0)	8 (14)	16 (17)	0 (0)	5 (17.2)	2 (5.6)		

[a]*Test of difference across groups (overall Chi-square)*

[b]*High priority score (1-2), mid priority score (3-5) and low priority score (6-8). Patients in the high priority group have the lowest severity of illness and are most likely to get critical care resources in periods of scarcity.*

[c]*1 point for acute SOFA score less than 6, 2 points for SOFA score between 6-9, 3 points for SOFA score 10-12, and 4 points for SOFA score greater than 12.*

[d] *On April 28th, 2020, institutions began to use an estimate of life expectancy instead of a measure of co-morbidities in response to the Massachusetts revised guidelines. Total patients with comorbidity scores are n=230; total patients with life expectancy scores are n=268. Column percentages for comorbidity points and life expectancy points were based on these totals.*

© 2022 Riviello ED et al. *JAMA Network Open.*

eTable 13. Detailed Scores Overall and by Race Among Patients With No COVID-19

		Race						
	Overall (n=273)	Black (n=22)	White (n=204)	Asian (n=5)	other (n=17)	unknown (n=28)	p-value[a] Black vs White	p-value[a] Black vs all others
Priority Group,[b] n (%)								
Low	13 (4.8)	1 (4.5)	10 (4.9)	0 (0)	1 (5.9)	1 (3.6)		
Mid	78 (28.6)	6 (27.3)	59 (28.9)	0 (0)	6 (35.3)	7 (25)		
High	182 (66.7)	15 (68.2)	135 (66.2)	0 (0)	10 (58.8)	20 (71.4)	0.98	0.97
SOFA group,[c] n (%)								
1- Least severe	221 (80.9)	17 (77.3)	165 (80.9)	1 (50)	14 (82.4)	24 (85.7)	0.74	0.7
2	34 (12.5)	4 (18.2)	25 (12.3)	1 (50)	2 (11.8)	2 (7.1)		
3	10 (3.7)	1 (4.5)	8 (3.9)	0 (0)	1 (5.9)	0 (0)		
4- Most severe	8 (2.9)	0 (0)	6 (2.9)	0 (0)	0 (0)	2 (7.1)		
Comorbidity points,[d] n (%)								
0-Least severe	80 (59.7)	5 (22.7)	50 (24.5)	1 (50)	9 (52.9)	15 (53.6)	0.93	0.78
2	41 (30.6)	4 (18.2)	31 (15.2)	0 (0)	2 (11.8)	4 (14.3)		
4-Most severe	13 (9.7)	1 (4.5)	10 (4.9)	0 (0)	0 (0)	2 (7.1)		
Life expectancy points,[d] n (%)								
0-Least severe	78 (56.1)	8 (36.4)	66 (32.4)	1 (50)	0 (0)	3 (10.7)	0.84	0.72
2	49 (35.2)	3 (13.6)	37 (18.1)	0 (0)	5 (29.4)	4 (14.3)		
4-Most severe	12 (8.6)	1 (4.5)	10 (4.9)	0 (0)	1 (5.9)	0 (0)		

[a]*Test of difference across groups (overall Chi-square)*

[b]*High priority score (1-2), mid priority score (3-5) and low priority score (6-8). Patients in the high priority group have the lowest severity of illness and are most likely to get critical care resources in periods of scarcity.*

[c]*1 point for acute SOFA score less than 6, 2 points for SOFA score between 6-9, 3 points for SOFA score 10-12, and 4 points for SOFA score greater than 12.*

[d] *On April 28th, 2020, institutions began to use an estimate of life expectancy instead of a measure of co-morbidities in response to the Massachusetts revised guidelines. Total patients with comorbidity scores are n=230; total patients with life expectancy scores are n=268. Column percentages for comorbidity points and life expectancy points were based on these totals.*

© 2022 Riviello ED et al. *JAMA Network Open.*

eTable 14. Priority Score Discrimination and Calibration for In-Hospital Mortality, Stratified by Race and Hispanic Ethnicity

	AUROC	Hosmer-Lemeshow test
All patients	0.79	0.19
Black	0.76	0.43
White	0.78	0.50
Asian	Too few to calculate	
other race	0.83	0.87
unknown race	0.82	0.82
Hispanic	0.82	0.04
non-Hispanic	0.79	0.70
unknown ethnicity	0.73	0.94
Socially vulnerable areas	0.81	0.37
Non-socially vulnerable areas	0.78	0.56

AUROC is area under the receiver operating characteristic.

© 2022 Riviello ED et al. *JAMA Network Open.*

eTable 15. Priority Score Discrimination and Calibration for In-Hospital Mortality, Stratified by Race and COVID-19 Status

	AUROC (ALL)	AUROC (COVID only)	AUROC (non-COVID only)
All patients	0.79	0.77	0.77
Black	0.76	0.78	0.58
White	0.78	0.71	0.8
Asian	Too few to calculate		
other race	0.83	0.83	0.76
unknown race	0.82	0.86	0.76

© 2022 Riviello ED et al. *JAMA Network Open.*

eTable 16. Estimated Excess Deaths by Race in Modelled Scenarios of Ventilator Scarcity

Panel A: <=1 get ventilator

Vents: Priority Score Allocation (cutoff: <= 1 get ventilator)						
Race	Total Number Ventilated	Excess Deaths with Priority Score	% Excess Deaths	p-value Black vs. White	p-value Black vs. all others	
Overall	244	90	36.9%			
Asian	7	2	28.6%			
Black	41	20	48.8%			
Other	26	8	30.8%			
Unknown	50	14	28.0%			
White	120	46	38.3%	0.24		
non-Black (all others)	203	70	34.5%		0.08	

Panel B: <=2 get ventilator

Vents: Priority Score Allocation (cutoff: <= 2 get ventilator)						
Race	Total Number Ventilated	Excess Deaths with Priority Score	% Excess Deaths	p-value Black vs. White	p-value Black vs. all others	
Overall	244	76	31.1%			
Asian	7	1	14.3%			
Black	41	18	43.9%			
Other	26	7	26.9%			
Unknown	50	10	20.0%			
White	120	40	33.3%	0.22		
non-Black (all others)	203	58	28.6%		0.05	

© 2022 Riviello ED et al. *JAMA Network Open.*

Panel C: <=3 get ventilator

Vents: Priority Score Allocation (cutoff: <= 3 get ventilator)						
Race	Total Number Ventilated	Excess Deaths with Priority Score	% Excess Deaths	p-value Black vs. White	p-value Black vs. all others	
Overall	244	40	16.4%			
Asian	7	0	0.0%			
Black	41	11	26.8%			
Other	26	4	15.4%			
Unknown	50	3	6.0%			
White	120	22	18.3%	0.25		
non-Black (all others)	203	29	14.3%		0.05	

Panel D: <= 4 get ventilator

Vents: Priority Score Allocation (cutoff: <=4 get ventilator)					
**Race**	**Total Number Ventilated**	**Excess Deaths with Priority Score**	**% Excess Deaths**	**p-value Black vs. White**	**p-value Black vs. all others**
Overall	244	27	11.1%		
Asian	7	0	0.0%		
Black	41	5	12.2%		
Other	26	4	15.4%		
Unknown	50	1	2.0%		
White	120	17	14.2%	0.75	
non-Black (all others)	203	22	10.8%		0.80

© 2022 Riviello ED et al. *JAMA Network Open.*

Panel E: <=5 get ventilator

Vents: Priority Score Allocation (cutoff: <= 5 get ventilator)						
Race	Total Number Ventilated	Excess Deaths with Priority Score	% Excess Deaths	p-value Black vs. White	p-value Black vs. all others	
Overall	244	8	3.3%			
Asian	7	0	0.0%			
Black	41	2	4.9%			
Other	26	1	3.8%			
Unknown	50	0	0.0%			
White	120	5	4.2%	0.85		
non-Black (all others)	203	6	3.0%		0.53	

Panel F: <=6 get ventilator

Vents: Priority Score Allocation (cutoff: <= 6 get ventilator)						
Race	Total Number Ventilated	Excess Deaths with Priority Score	% Excess Deaths	p-value Black vs. White	p-value Black vs. all others	
Overall	244	0	0.0%			
Asian	7	1	14.3%			
Black	41	0	0.0%			
Other	26	0	0.0%			
Unknown	50	0	0.0%			
White	120	0	0.0%	-		
non-Black (all others)	203	0	0.0%		-	

Panel G: <=7 get ventilator

© 2022 Riviello ED et al. *JAMA Network Open.*

Vents: Priority Score Allocation (cutoff: <= 7 get ventilator)						
Race	Total Number Ventilated	Excess Deaths with Priority Score	% Excess Deaths	p-value Black vs. White	p-value Black vs. all others	
Overall	244	0	0.0%			
Asian	7	0	0.0%			
Black	41	0	0.0%			
Other	26	0	0.0%			
Unknown	50	0	0.0%			
White	120	0	0.0%	-		
non-Black (all others)	203	0	0.0%		-	

© 2022 Riviello ED et al. *JAMA Network Open.*

eTable 17. Estimated Excess In-Hospital Deaths by Priority Score vs Random Allocation
Panel A: <=1 get ventilator

Vents: Priority Score vs. Random (cutoff: <= 1 get ventilator)										
	Real Data			Priority Score				Random Allocation		p-value comparing priority and random allocation
Race	Total Vented	Total Vented and Survived	Total Vented in Scores 2+	Died but would have survived if they received a vent		Died with a vent		Died but would have survived if they received a vent (out of 10,000 trials)	Died with a vent (out of 10,000 trials)	
	n	n	n	n	%	n	%	%	%	
Asian	7	5	4	2	28.57%	2	28.57%	51.77%	7.81%	0.11
Black	41	26	33	20	48.78%	13	31.71%	46.06%	10.08%	0.64
Other	26	18	14	8	30.77%	6	23.08%	50.23%	8.56%	0.02
Unknown	50	33	29	14	28.00%	15	30.00%	47.92%	9.35%	0.002
White	120	81	78	46	38.33%	32	26.67%	48.97%	8.92%	0.01
Total	244	163	158	90	36.89%	68	27.87%	48.48%	9.13%	< 0.001

Panel B: <=2 get ventilator

Vents: Priority Score vs. Random Allocation (cutoff: <= 2 get ventilator)										
	Real Data			Priority Score				Random Allocation		p-value comparing priority and random allocation
Race	Total Vented	Total Vented and Survived	Total Vented in Scores 3+	Died but would have survived if they received a vent		Died with a vent		Died but would have survived if they received a vent (out of 10,000 trials)	Died with a vent (out of 10,000 trials)	
	n	n	n	n	%	n	%	%	%	
Asian	7	5	3	1	14.29%	2	28.57%	38.47%	13.05%	0.09
Black	41	26	30	18	43.90%	12	29.27%	34.06%	16.96%	0.91
Other	26	18	13	7	26.92%	6	23.08%	37.14%	14.27%	0.14
Unknown	50	33	23	10	20.00%	13	26.00%	35.31%	15.81%	0.01
White	120	81	71	40	33.33%	31	25.83%	36.29%	15.02%	0.25
Total	244	163	140	76	31.15%	64	26.23%	35.87%	15.37%	0.06

© 2022 Riviello ED et al. *JAMA Network Open.*

Panel C: <=3 get ventilator

Vents: Priority Score vs. Random Allocation (cutoff: <= 3 get ventilator)										
	Real Data			Priority Score				Random Allocation		p-value comparing priority and random allocation
Race	Total Vented	Total Vented and Survived	Total Vented in Scores 4+	Died but would have survived if they received a vent		Died with a vent		Died but would have survived if they received a vent (out of 10,000 trials)	Died with a vent (out of 10,000 trials)	
	n	n	n	n	%	n	%	%	%	
Asian	7	5	1	0	0.00%	1	14.29%	26.05%	18.05%	0.06
Black	41	26	19	11	26.83%	8	19.51%	23.16%	23.32%	0.71
Other	26	18	10	4	15.38%	6	23.08%	25.42%	19.52%	0.12
Unknown	50	33	11	3	6.00%	8	16.00%	24.00%	21.61%	0.001
White	120	81	44	22	18.33%	22	18.33%	24.59%	20.62%	0.06
Total	244	163	85	40	16.39%	45	18.44%	24.36%	21.08%	0.002

Panel D: <= 4 get ventilator

Vents: Priority Score vs. Random Allocation (cutoff: <= 4 get ventilator)										
	Real Data			Priority Score				Random Allocation		p-value comparing priority and random allocation
Race	Total Vented	Total Vented and Survived	Total Vented in Scores 5+	Died but would have survived if they received a vent		Died with a vent		Died but would have survived if they received a vent (out of 10,000 trials)	Died with a vent (out of 10,000 trials)	
	n	n	n	n	%	n	%	%	%	
Asian	7	5	0	0	0.00%	0	0.00%	17.22%	21.77%	0.11
Black	41	26	13	5	12.20%	8	19.51%	15.30%	27.75%	0.29
Other	26	18	9	4	15.38%	5	19.23%	16.87%	23.39%	0.42
Unknown	50	33	6	1	2.00%	5	10.00%	15.91%	25.74%	0.004
White	120	81	35	17	14.17%	18	15.00%	16.34%	24.65%	0.26
Total	244	163	63	27	11.07%	36	14.75%	16.16%	25.18%	0.02

© 2022 Riviello ED et al. *JAMA Network Open.*

Panel E: <=5 get ventilator

Vents: Priority Score vs. Random Allocation (cutoff: <= 5 get ventilator)										
	Real Data			Priority Score				Random Allocation		
Race	Total Vented	Total Vented and Survived	Total Vented in Scores 6+	Died but would have survived if they received a vent		Died with a vent		Died but would have survived if they received a vent (out of 10,000 trials)	Died with a vent (out of 10,000 trials)	p-value comparing priority and random allocation
	n	n	n	n	%	n	%	%	%	
Asian	7	5	0	0	0.00%	0	0.00%	10.33%	24.50%	0.18
Black	41	26	8	2	4.88%	6	14.63%	9.10%	31.32%	0.17
Other	26	18	5	1	3.85%	4	15.38%	9.95%	26.36%	0.15
Unknown	50	33	1	0	0.00%	1	2.00%	9.47%	29.12%	0.01
White	120	81	16	5	4.17%	11	9.17%	9.68%	27.86%	0.02
Total	244	163	30	8	3.28%	22	9.02%	9.59%	28.44%	< 0.001

Panel F: <=6 get ventilator

Vents: Priority Score vs. Random Allocation (cutoff: <= 6 get ventilator)										
	Real Data			Priority Score				Random Allocation		
Race	Total Vented	Total Vented and Survived	Total Vented in Scores 7+	Died but would have survived if they received a vent		Died with a vent		Died but would have survived if they received a vent (out of 10,000 trials)	Died with a vent (out of 10,000 trials)	p-value comparing priority and random allocation
	n	n	n	n	%	n	%	%	%	
Asian	7	5	0	0	0.00%	0	0.00%	4.41%	26.78%	0.28
Black	41	26	4	1	2.44%	3	7.32%	3.88%	34.34%	0.32
Other	26	18	0	0	0.00%	0	0.00%	4.26%	28.86%	0.14
Unknown	50	33	1	0	0.00%	1	2.00%	4.07%	31.92%	0.07
White	120	81	3	0	0.00%	3	2.50%	4.15%	30.51%	0.01
Total	244	163	8	1	0.41%	7	2.87%	4.11%	31.16%	0.002

© 2022 Riviello ED et al. *JAMA Network Open.*

Panel G: <=7 get ventilator

Vents: Priority Score vs. Random Allocation (cutoff: <= 7 get ventilator)										
	Real Data			Priority Score				Random Allocation		p-value comparing priority and random allocation
Race	Total Vented	Total Vented and Survived	Total Vented in Score 8	Died but would have survived if they received a vent		Died with a vent		Died but would have survived if they received a vent (out of 10,000 trials)	Died with a vent (out of 10,000 trials)	
	n	n	n	n	%	n	%	%	%	
Asian	7	5	0	0	0.00%	0	0.00%	3.61%	27.23%	0.30
Black	41	26	2	0	0.00%	2	4.88%	3.07%	34.75%	0.13
Other	26	18	0	0	0.00%	0	0.00%	3.37%	29.29%	0.17
Unknown	50	33	1	0	0.00%	1	2.00%	3.24%	32.33%	0.10
White	120	81	1	0	0.00%	1	0.83%	3.61%	30.90%	0.02
Total	244	163	4	0	0.00%	4	1.64%	3.28%	31.56%	0.002

© 2022 Riviello ED et al. *JAMA Network Open.*

eFigure 1. Study Cohort

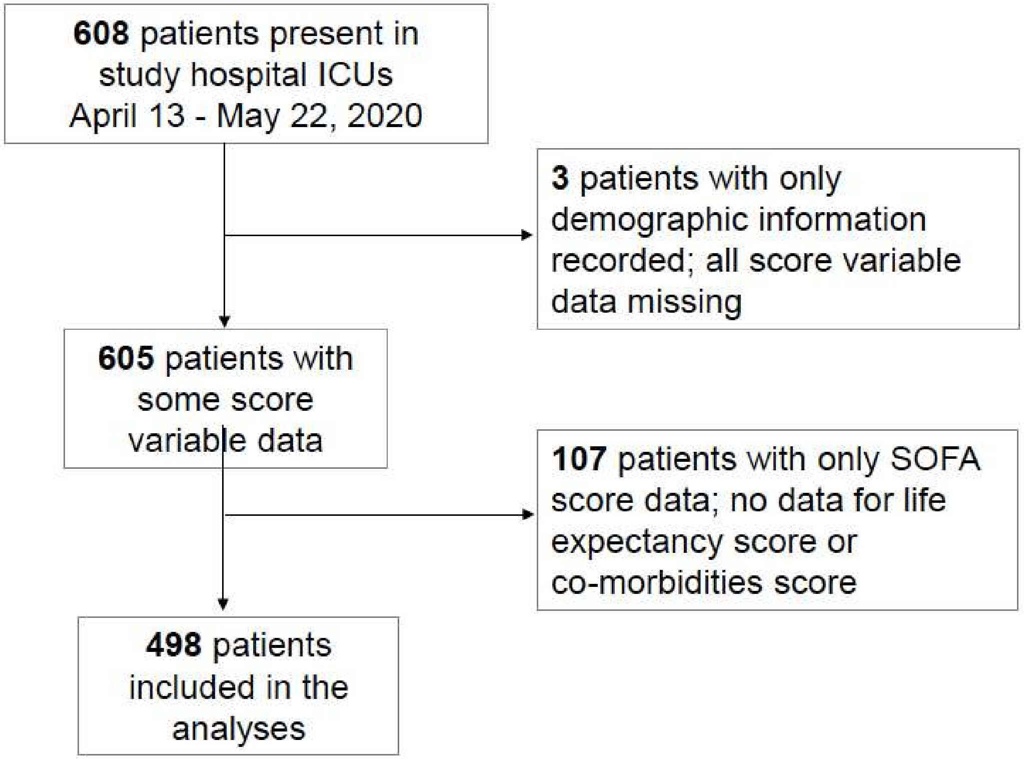

© 2022 Riviello ED et al. *JAMA Network Open.*

eFigure 2. AUROCs or In-Hospital Mortality for All Patients (A), Black Patients (B), White Patients (C), Hispanic Patients (D), Non-Hispanic Patients (E), Patients From Socially Vulnerable Areas (F), and Patients From Non–Socially Vulnerable Areas

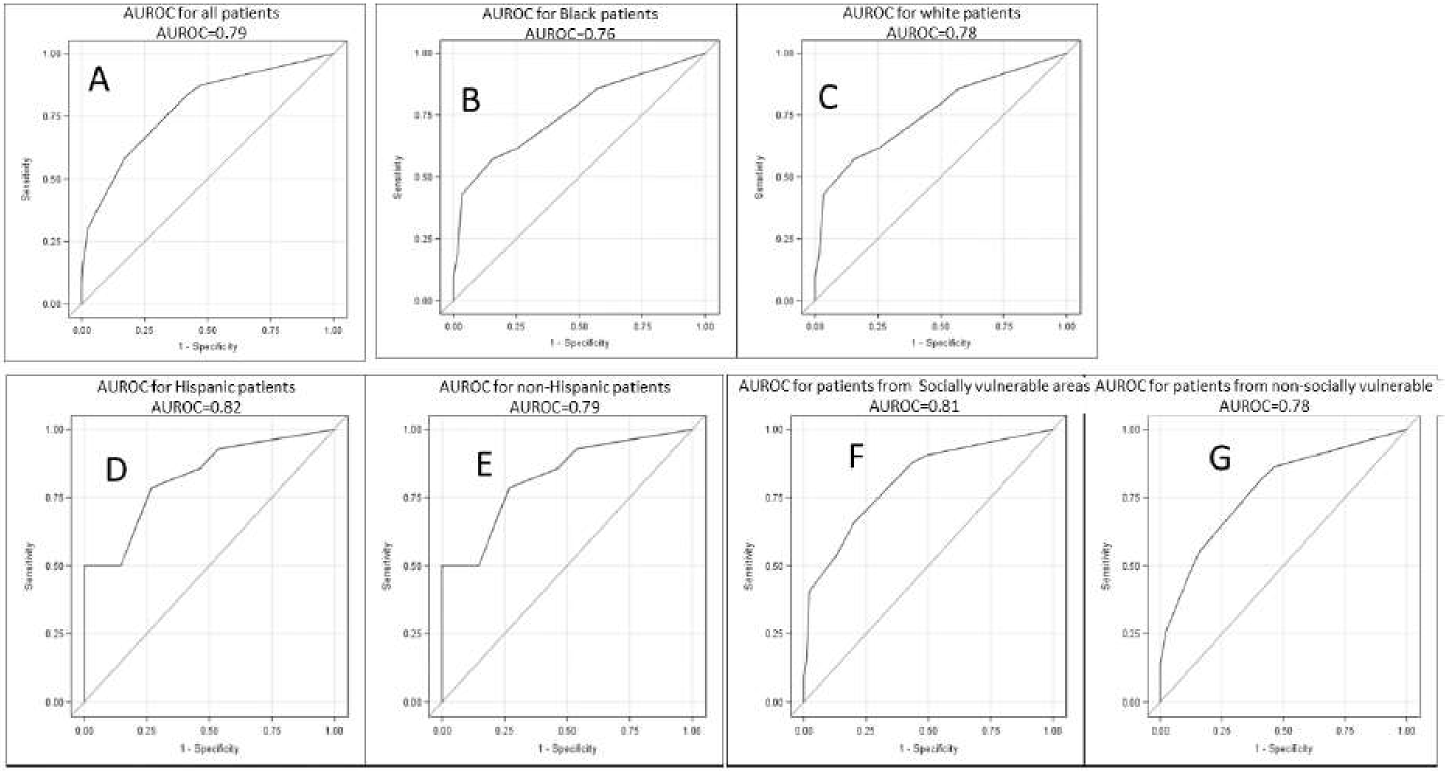

© 2022 Riviello ED et al. *JAMA Network Open.*

eFigure 3. In-Hospital Mortality by Priority Score for White vs Asian Patients (A), White vs Other Race (B), White vs Unknown Race (C), and Hispanic vs Non-Hispanic Patients (D)

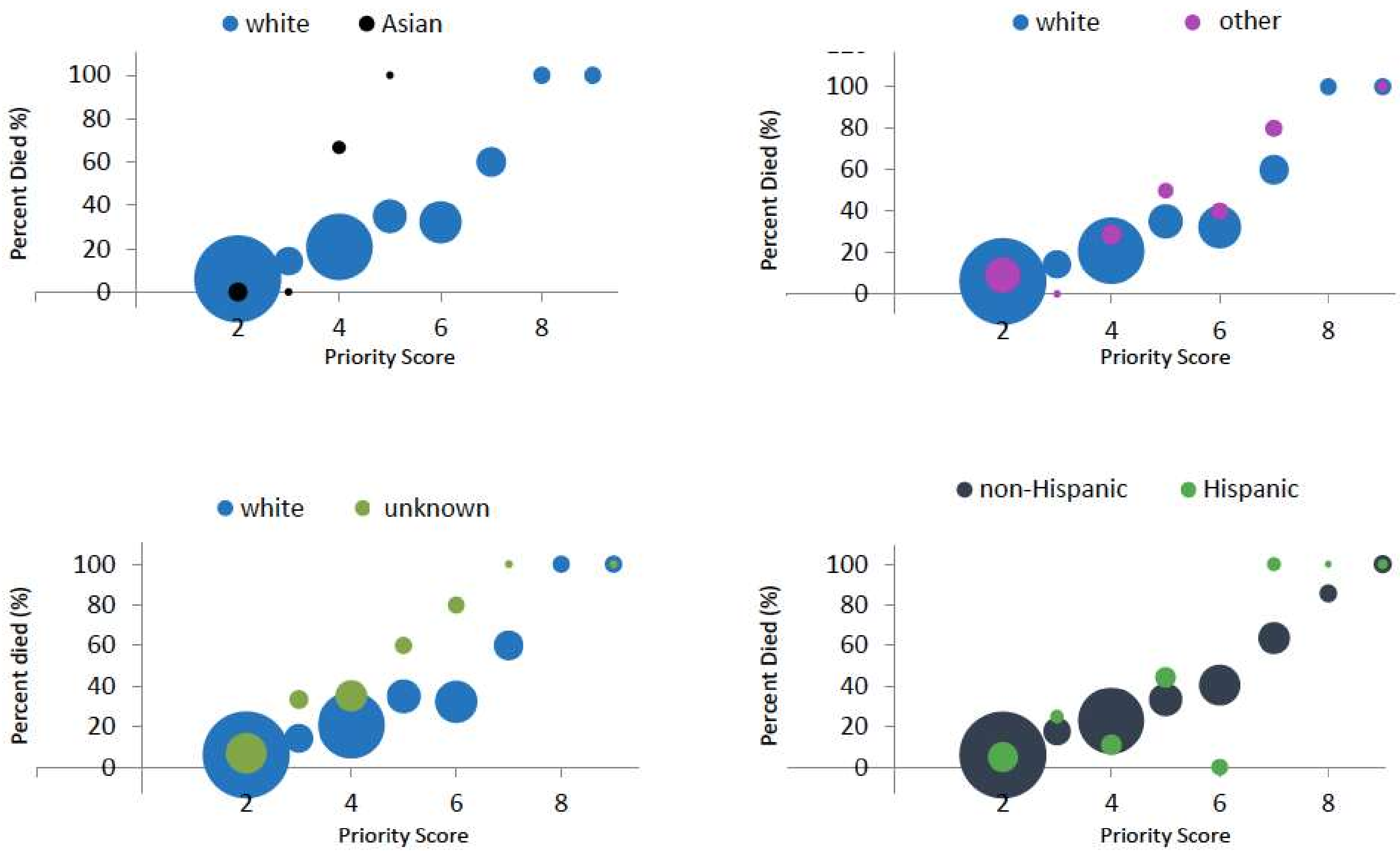

The size of the bubble represents the total number of patients in each priority score group.

© 2022 Riviello ED et al. *JAMA Network Open.*

eFigure 4. In-Hospital Mortality by Priority Score for Patients From Socially Vulnerable Areas and Non–Socially Vulnerable Areas

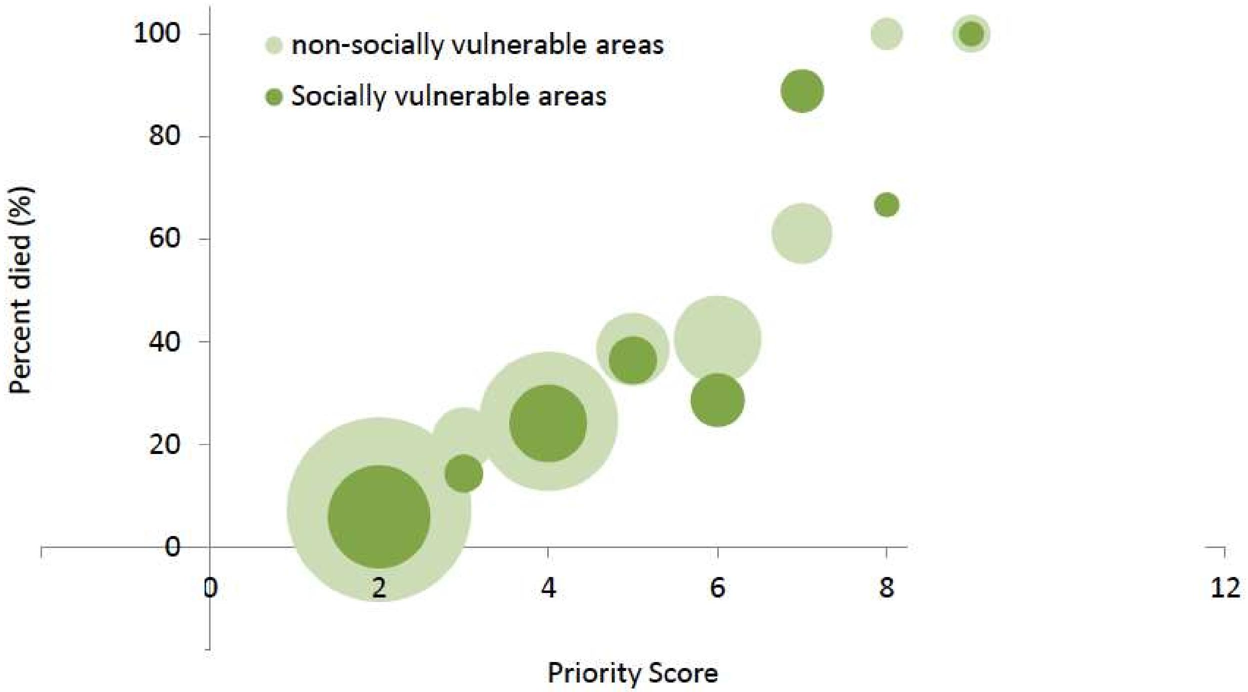

The size of the bubble represents the total number of patients in each priority score group.

© 2022 Riviello ED et al. *JAMA Network Open.*
